# Extraction, Structural, and Antioxidant Properties of Oligosaccharides Hydrolyzed from *Panax notoginseng* by Ultrasonic-Assisted Fenton Degradation

**DOI:** 10.3390/ijms24054506

**Published:** 2023-02-24

**Authors:** Xiaoyan Xu, Guanfeng Deng, Xiao Li, Pingjin Li, Tao Chen, Lijun Zhou, Yan Huang, Ming Yuan, Chunbang Ding, Shiling Feng

**Affiliations:** College of Life Science, Sichuan Agricultural University, Ya’an 625014, China

**Keywords:** *Panax notoginseng*, polysaccharide, Fenton degradation

## Abstract

Plant polysaccharides exhibit many biological activities that are remarkably affected by molecular size and structures. This study aimed to investigate the degradation effect of ultrasonic-assisted Fenton reaction on the *Panax notoginseng* polysaccharide (PP). PP and its three degradation products (PP3, PP5, and PP7) were obtained from optimized hot water extraction and different Fenton reaction treatments, respectively. The results showed that the molecular weight (Mw) of the degraded fractions significantly decreased after treatment with the Fenton reaction. But the backbone characteristics and conformational structure were similar between PP and PP-degraded products, which was estimated by comparing monosaccharides composition, functional group signals in FT-IR spectra, X-ray differential patterns, and proton signals in ^1^H NMR. In addition, PP7, with an Mw of 5.89 kDa, exhibited stronger antioxidant activities in both the chemiluminescence-based and HHL5 cell-based methods. The results indicated that ultrasonic-assisted Fenton degradation might be used to improve the biological activities of natural polysaccharides by adjusting the molecular size.

## 1. Introduction

*Panax notoginseng* (Burk.) F. H. Chen is a traditional Chinese medicinal and edible plant belonging to the *Araliaceae* ginseng species. The roots of *P. notoginseng*, known as Sanqi or Tianqi in Chinese herbal medicine, have been used as a tonic and anti-hemorrhagic drug for more than 600 years in east Asian countries. The pharmacological effects of Sanqi are mainly attributed to the notoginseng saponins, which exhibited remarkable hemostatic, anti-inflammatory, and anti-tumor effects, etc. [1]. Besides the roots, the aerial parts are also used as functional foods and dietary supplements. Their health-beneficial effects may be due to various active substances, including polysaccharides, flavonoids, volatile compounds, etc. [2,3]. Our previous studies demonstrated that *P. notoginseng* polysaccharides were potential in vivo redox homeostasis stabilizers. *P. notoginseng* polysaccharides could efficiently restore disrupted redox balance by triggering an endogenous antioxidant defense system in model animal *Caenorhabditis elegans*, which improves the stress resistance and healthspan of the worms [2,4]. In addition, the hepatoprotective, immunomodulatory, and neuroprotective effects of *P. notoginseng* polysaccharides were also reported in other studies [5,6,7]. These results indicated that polysaccharides might be the primary compounds contributing to the health-beneficial effects of *P. notoginseng*.

The majority of polysaccharides extracted from *P. notoginseng* is pectin, including arabinogalactan (AG), rhamnogalacturonan (RG), and homogalacturonan (HG), etc. [8,9,10]. The activities of polysaccharides are highly affected by the molecular weight, glycosidic bond type, and monosaccharide composition [11,12,13]. Numerous studies have shown that low polymerization polysaccharides with high RG-I content have better performance in anti-cancer and anti-oxidation effects [14,15]. Reducing the polymerization of polysaccharides can be obtained by acid hydrolysis or enzymatic hydrolysis. However, acid treatment leads to the excessive hydrolysis of the glycosidic bond between galacturonic acid and rhamnose residues in the RG-I region. The application of enzymatic treatments is limited by the cleavage sites and cost. Therefore, new approaches to efficiently change the chain structures and activities of polysaccharides are explored, like Fenton degradation. Fenton reaction uses ferrous ions as a catalyst and hydrogen peroxide to generate OH free radicals. These free radicals can react with C bonds and H atoms, destroy glycosidic bonds, and then depolymerize polysaccharides. Li et al. employed the Fenton reaction to generate low molecular polysaccharides with improved antioxidant activities from citrus pectin [16]. The antioxidant and anti-inflammatory activities and prebiotic capacity of okra pectin were enhanced by the Fenton reaction [15]. This reaction turns out to be an efficient method to depolymerize polysaccharides with the advantages of energy saving, environmental protection, and mild reaction conditions.

In recent years, some new extract methods have been used to improve the yield of plant polysaccharides, such as CO_2_ supercritical fluid extraction, ultrasound-assisted extraction, microwave-assisted extraction, and acid/alkali-assisted extraction [17]. However, these new methods also presented some disadvantages, for instance, high energy consumption, environmental damage, and the demolition of polysaccharide polymers. Hence, hot water extraction, the classical extraction method, is still wildly used to extract plant polysaccharides due to the low cost, easy operation, simple equipment, and the preservation of original chain conformation, in spite of the limitation of low extraction yield [18]. In order to achieve better extraction efficiency, the response surface methodology (RSM) has been applied to optimize the hot water extraction process. This technique only takes a short period of time to integrate the independent variables, identify interrelationships between variables, and develop a suitable experimental design to explore the maximum extraction efficiency [19]. In this study, we extracted polysaccharide (PP) from the leaves of *P. notoginseng*. The extraction was optimized by a single-factor test and RSM. PP3, PP5, and PP7 were generated by hydrolyzing from PP in different Fenton reaction systems. The structures of *P. notoginseng* polysaccharides were revealed, and the antioxidant activities were evaluated.

## 2. Results and Discussion

### 2.1. Optimization of Extraction Conditions

#### 2.1.1. Single Factors Assay

To improve the extraction, we first analyzed the effects of temperature, liquid:solid ratio, and extraction time on the extraction yields of PP. As shown in Supplementary Figure S1a, the yield of PP increased gradually with the temperature increase from 50 to 85 °C, but when the temperature exceeded 85 °C, the yield declined. This result might be due to the destruction of the structure of PP at high temperatures. As shown in Supplementary Figure S1b, when the extraction time was extended from 0.5 to 1.5 h, the polysaccharide yield showed an upward trend, but there was no significant difference after 1.5 h. As shown in Supplementary Figure S1c, the polysaccharide yield increased gradually when the liquid:solid ratio was from 10 to 15 mL/g, and the yield reached the maximum at 15 mL/g. Considering the preservation of polysaccharide structure and the maintenance of high extraction efficiency, the RSM was performed with 85 °C, 1.5 h, and 15 mL/g as the center point.

#### 2.1.2. ANOVA Analysis of RSM

Based on the results of single factors, Design-Expert 10 software was applied to perform multiple regression analysis. The results of the responses (yield) in the factorial design are presented in Table 1, and the full-variable quadratic regression equation was obtained:

(1) $$Y=11.55+0.74A+0.28B\;-\;0.25C+0.33\mathrm{AB}\;-\;0.16\mathrm{AC}\;-\;0.33\mathrm{BC}\;-\;0{.85A}^{2}\;-\;1{.24B}^{2}\;-\;1{.51C}^{2}$$

The results of the response surface analysis of variance are shown in Table 2. The F value of significance analysis in the model was 9.13, and the corresponding probability value was *p* < 0.01, indicating that the model was highly significant. The mismatch term was 0.1058 (*p* > 0.05), indicating that its relative pure error was not significant. The coefficient of determination R^2^ was 0.9215, suggesting that 92.15% of yield variation was attributed to variable factors. A low coefficient variation (CV% = 2.58%) indicated that the model fitted well with the quadratic model based on analysis of variance. Therefore, the model could be used to predict the best conditions for extracting PP using hot water. According to the F-value and *p*-value, the influence of each single factor on the yield of PP was as follows: temperature (*A*) > time (*B*) > liquid:material ratio (*C*).

#### 2.1.3. Analysis of Response Surface Plot and Contour Plot

**Figure 1:**
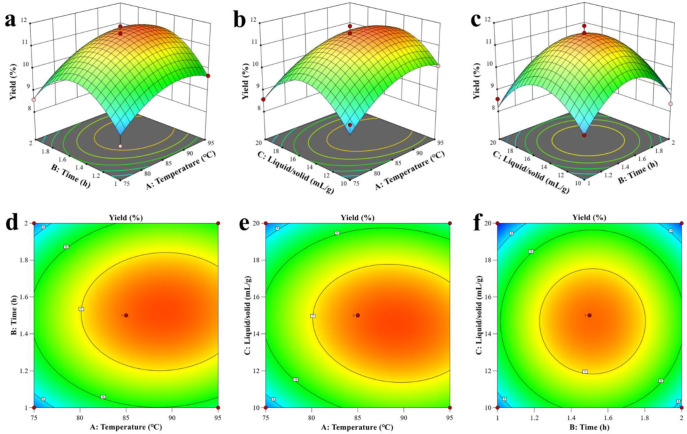
Results of response surface analysis. Response surface plots of the interactions between extraction temperature and time. (**a**) Extraction temperature and liquid:solid ratio. (**b**) Extraction time and liquid:solid ratio. (**c**) A, B, and C represent the extraction temperature, extraction time, and liquid:solid ratio, and (**d**–**f**) are their contour plots, respectively.

The response surface and contour plots are a graphical representation of the regression equation of selected variables. According to the steepness of the response surface diagram, the effect of independent variables can be directly reflected in the response variable [20]. As shown in Figure 1, the interactive curved surfaces (Figure 1a–c) between time/temperature, liquid-ratio/temperature, and time/liquid-ratio were similar, and the contour plots (Figure 1d–f) were all manifested as oval shapes, indicating that the interaction between them displayed notable effects on the response value. Based on the regression model, the best extraction parameters were: 89.84 °C, 1.60 h, and 14.36 mL/g, respectively. Taking practical conditions into consideration, the parameters were adjusted to: 90.0 °C, 1.60 h, 14.00 mL/g. Under the three parallel verification tests, the average yield of PP was 11.30% ± 0.73%, which was similar to the predicted yield of 11.78%. These results suggested that the optimized extraction process was reliable and suitable for the extraction of PP. Wang et al. employed high-pressure extraction to gain a higher yield of *P. notoginseng* polysaccharide [21]. Additionally, ultrasound-assisted extraction has also been used to extract *P. notoginseng* polysaccharides, but the yields ranged from 3.3% to 13.36% [6,7,22]. Those methods dramatically affected both the yields and the structure of polysaccharides and further led to the various. Therefore, this study applied the RSM method in the optimization process of hot-water extraction to improve the yield of *P. notoginseng* polysaccharide.

### 2.2. Chemical Composition of P. notoginseng Polysaccharides

As shown in Table 3, the yields of PP3, PP5, and PP7 were 27.70 ± 0.65%, 32.89 ± 1.89%, and 24.74 ± 0.54% after degradation by the Fenton reaction [23]. The neutral sugar content of PP, PP3, PP5, and PP7 were 73.32 ± 3.15%, 81.63 ± 2.72%, 84.29 ± 2.98%, and 85.82 ± 2.15%, respectively. Both protein content and polyphenol in degraded products of PP were significantly decreased. Compared to PP, the protein content of PP3, PP5, and PP7 decreased by 41.64%, 38.46%, and 27.32%, respectively. Moreover, after the Fenton reaction, the polyphenol content of degraded polysaccharides was decreased from 8.72 ± 0.14% (PP) to 2.50 ± 0.05% (PP3), 1.96 ± 0.06% (PP5), and 1.39 ± 0.16%(PP7), respectively.

### 2.3. Structural Properties

#### 2.3.1. Molecular Weight Distribution and Configuration

**Figure 2:**
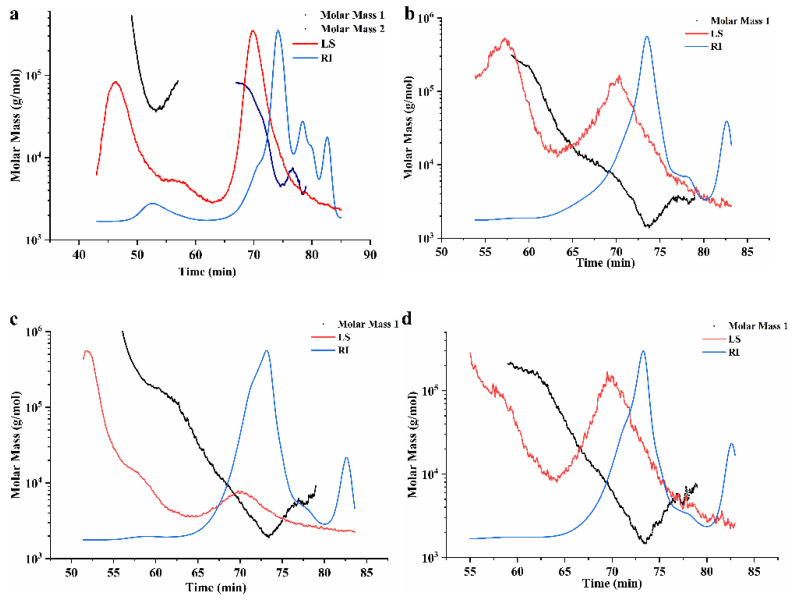
High-performance size exclusion chromatograms of *P. notoginseng* polysaccharides. The LS refers to the multi-angle laser light scattering signal; RI is the indication difference signal; Molar mass is the molecular weight fitted by two signals. The molecular distribution of PP (**a**), PP3 (**b**), PP5 (**c**), and PP7 (**d**).

The molecular weight and chain conformation of PP and its degradation products are shown in Figure 2. PP demonstrated two peaks, and the first peak accounted for 8.40% of the total peak area with an average molecular weight (Mw) of 64.69 kDa. Another peak with Mw of 14.64 kDa accounted for 91.60% of the total peak area. Three oligosaccharides after the Fenton reaction had only one peak, and the molecular weight ranged from 5.89 to 8.92 kDa. The molecular weight and chain conformation of polysaccharides significantly affected their biological activities and potential applications [24]. Hence, it is crucial to find methods to adjust the molecular weight of polysaccharides without modifying the configuration [25]. Zhang et al. obtained three kinds of pectin oligosaccharides from citrus peel with Mw in the range of 3000~4000 Da, 3000~2000 Da and less than 2000 Da by adjusting the concentration of trifluoroacetic acid [26], Combo et al. obtained beet pectin polysaccharide with molecular weight less than 8 kDa by enzymatic hydrolysis [27]. Our study confirmed that the molecular weight of PP could be degraded to about 5.5–9 kDa by adjusting the concentration of ferrous sulfate at the same reaction time. According to the experimental results, except for the first peak of PP, the radius of gyration (Rg) of the second peak of PP and the degradation products of PP were less than 30 nm, which was related to the Mw and compact configuration of these degradation products. In addition, it might also be related to the shrinkage of molecules caused by polysaccharides in a salt solution. The molecular configuration of polymers can be divided into rods, irregular threads, and spheres [11]. As shown in Figure 2a, the PP displayed a “U-shaped” curve with the molar mass as the X-axis, and the root mean square radius as the Y-axis, indicating that PP was a spherical polysaccharide with rich branched chains [28]. The k-values of the PP3, PP5, and PP7 products were −0.01, −0.04, and −0.06, respectively, indicating that the degraded polysaccharides also formed spherical conformation after the Fenton reaction.

#### 2.3.2. Monosaccharide Compositions

**Figure 3:**
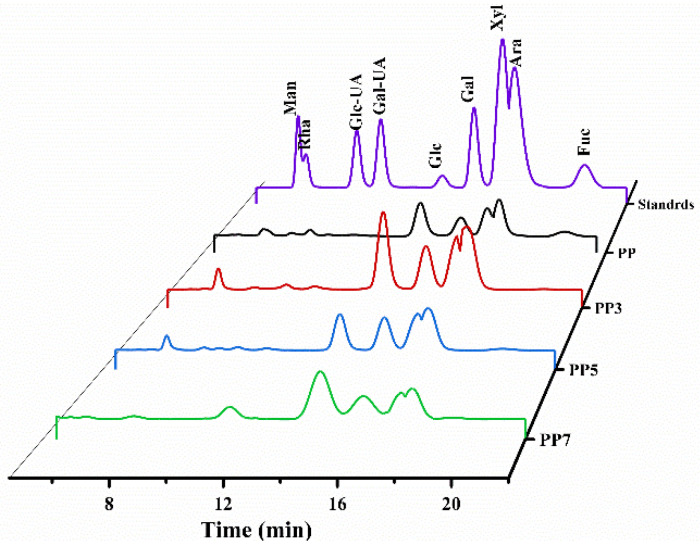
High-performance liquid chromatogram profiles of compositional monosaccharides of PP, PP3, PP5, and PP7.

Monosaccharide composition significantly affects the functional properties of polysaccharides. As shown in Table 3 and Figure 3, the four polysaccharides were composed of mannose (Man), glucose (Glc), galactose (Gal), xylose (Xyl), arabinose (Ara), and fucose (Fuc). The Glc, Gal, Xyl, and Ara were the main sugar units comprising more than 70% neutral sugar. And the ratio of those four sugar residues was similar in all four samples. However, the Fuc was removed in the degradation of PP, suggesting that Fuc residues were in the branch chain of PP and susceptible to Fenton degradation. Okra oligosaccharides degraded by the ultrasonic-assisted Fenton reaction exhibited similar results that the sugar residues in the branch chain were the main target of Fenton degradation [11].

#### 2.3.3. Fourier-Transform Infrared Spectroscopy

**Figure 4:**
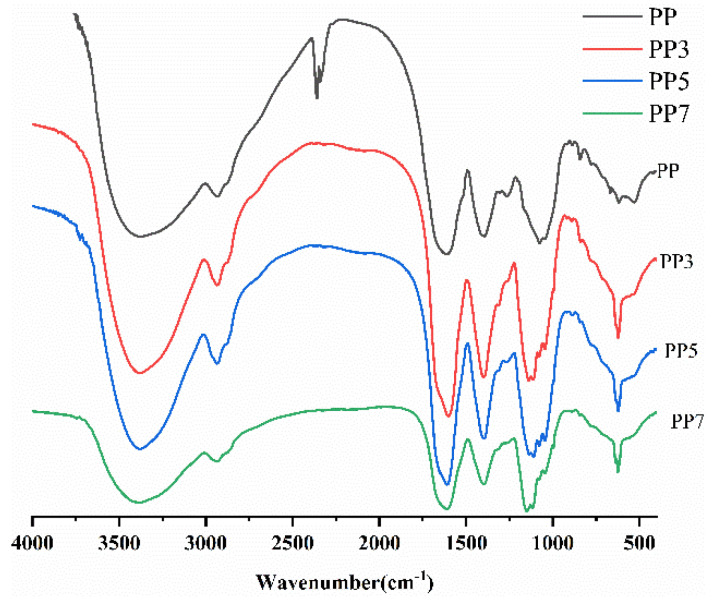
FT-IR spectra of PP, PP3, PP5, and PP7.

The FT-IR spectra of PP and its three oligosaccharides after the Fenton reaction are shown in Figure 4. Both PP and its depolymerized products displayed similar spectral bands. The major absorption around 3410 cm^−1^ in four polysaccharides was attributed to the stretching of hydroxyl groups, and the bands around 2930 cm^−1^ were attributed to the C–H stretching of CH_2_ groups. The absorption around 1620 cm^−1^ corresponds to the COO- stretching vibration of esterified groups, indicating the presence of uronic acids. The bands around 1420 cm^−1^ and 1140 cm^−1^ were owing to the bending vibration of C―H and C–O–C. The degree of methylation (DM) can be calculated by dividing the carboxylic ester and carboxylic acid group signals by the carboxylic ester signal. In this study, the DM values of PP, PP3, PP5, and PP7 were 24.6%, 17.9%, 34.3%, and 28.9%, respectively, indicating that the four samples were low methoxylated pectins.

#### 2.3.4. X-ray Diffraction

**Figure 5:**
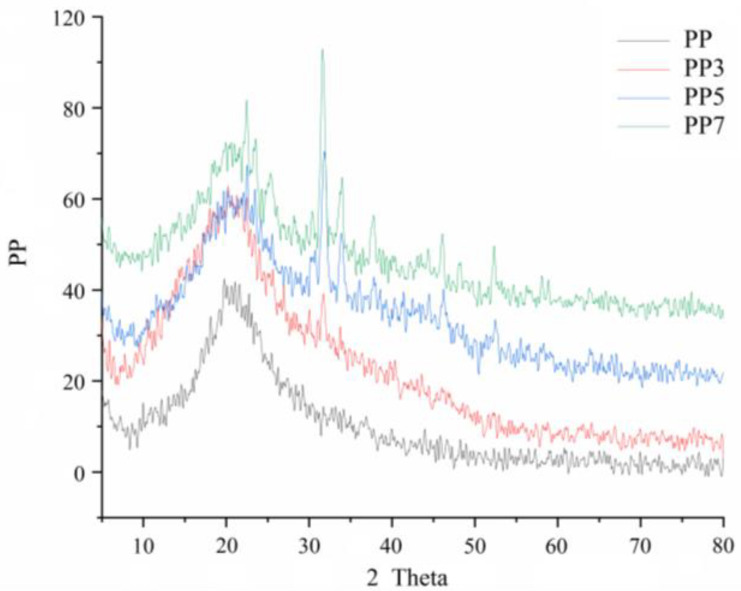
X-ray diffraction pattern of PP, PP3, PP5, and PP7.

XRD was used to demonstrate the amorphous or crystalline structure of the *P. notoginseng* polysaccharides. As shown in Figure 5, their characteristic peaks were similar, which were, at 2θ, equal to 19.1, 21.7, 24.5, 25.3, 32.4, 35.0, 39.2, and 47.1°, indicating similar crystal structures. Compared to PP, its degradation products displayed sharp peaks at 24.5, 32.4, and 35.0°, which might be due to the ultrasound treatment or the decrease in molecular weight [29]. These results indicated that the Fenton degradation reaction slightly affected the structure of PP.

#### 2.3.5. ^1^H NMR

To further estimated the structure change after the Fenton reaction, the ^1^H NMR spectra of four polysaccharides were employed to elucidate the hydrogen signal shift. As shown in Supplementary Figure S2, their anomeric proton signal was similar, distributing from 5.27 to 4.5 ppm. The signal at 5.25 ppm might be assigned to H-1 of Ara, and other signals for anomeric proton in 5.09, 4.95, and 4.83 might be H-1 of Gal, Glc, and Xyl, indicating that the backbones of the polysaccharides were not modified during Fenton reaction [11,30]. The major difference in the ^1^H NMR spectra was in the H-2 to H-5 area; the signal ranged from 4.10 to 3.01. Compared to the degradation product of PP, PP showed complicated signals, which might be due to the large number of branch chains. These results were in accordance with the Mw and monosaccharide composition change, which suggested that the Fenton reaction mainly degraded the branch chain of PP.

#### 2.3.6. Thermal Analysis

**Figure 6:**
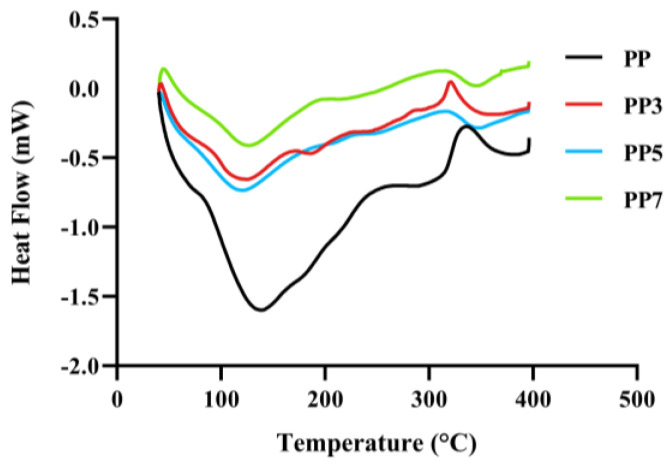
Differential scanning calorimetry (DSC) analysis of PP, PP3, PP5, and PP7.

Differential scanning calorimetry (DSC) was applied to reveal the thermal behavior of *P. notoginseng* polysaccharides and further to estimate how this behavior can be affected after the Fenton reaction. As shown in Figure 6, there was one endothermic peak and one exothermic peak among the four polysaccharides. The maximum endothermic peak of PP and its degradation product polysaccharide ranged from 112 °C to 139 °C, which might be related to the movement of bound water. The maximum exothermic peak of the four polysaccharides appeared at about 310–320 °C, which might be due to the chemical bond breaking of the polysaccharide. This indicated that all four polysaccharides might have good and similar thermal stability.

### 2.4. Antioxidant Activities of P. notoginseng Polysaccharides

#### 2.4.1. DPPH· Scavenging Abilities of *P. notoginseng* Polysaccharides

**Figure 7:**
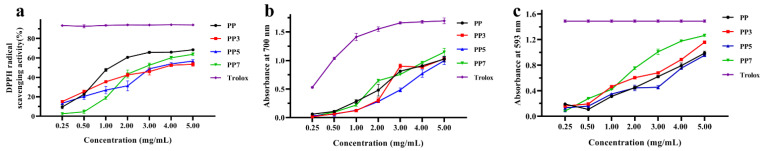
In vitro antioxidant activities of *P. notoginseng* polysaccharides at different concentrations. DPPH· scavenging abilities of PP, PP3, PP5, and PP7 (**a**), ferrous ion reduction force of PP, PP3, PP5, and PP7 (**b**), and total antioxidant capacities of PP, PP3, PP5, and PP7 (**c**).

DPPH is a stable organic free radical that has been widely used to identify the free radical scavenging ability of polysaccharides. As shown in Figure 7a, the four polysaccharides showed dose-dependent scavenging ability in the test concentration range. At the same concentration, the free radical scavenging power of crude polysaccharides was stronger than those of PP3, PP5, and PP7, which might be due to the higher polyphenol content of PP. The reducing hemiacetal hydroxyl contained in plant polysaccharides can absorb electrons, block the chain reaction of free radicals, prevent the formation of free radicals, or directly react with peroxides and reactive oxygen species to remove free radicals [31]. The half maximal inhibitory concentration (IC_50_) of PP3, PP5, and PP7 were 3.74, 3.25, and 2.69 mg/mL, respectively. PP7 had higher DPPH· scavenging ability than those of PP3 and PP5 due to the lower molecular weights, which was in accord with the results of the Chang group [32]. Wu et al. also showed that the antioxidant activities of polysaccharides are highly affected by molecular weight [11].

#### 2.4.2. Ferrous Ion Reduction Force of *P. notoginseng* Polysaccharides

The determination of reducing power takes the amount of Prussian blue Fe_4_ (Fe (CN)_6_) as the index. The antioxidant can reduce potassium ferricyanide and then use ferrous ions to form Prussian blue. This substance has the maximum absorption peak at 700 nm. The higher the absorbance value, the stronger the reducing power of the sample. The reducing capacity of polysaccharides was related to the molecular weight and monosaccharide composition [11]. 11As shown in Figure 7b, the reducing power of PP and its degradation products were dose-dependent. Compared to Trolox, the reducing power of these four polysaccharides was weak. PP7 exhibited better ferrous ion reduction power with half maximal effective concentration (EC_50_) of 2.95 mg/mL, compared to the EC_50_ of 3.46 mg/mL PP. The oligosaccharides with a non-compact structure have more free hydroxyl groups and surface area that more easily interact with ferrous ions. The result was similar to the okra polysaccharide and its oligosaccharide, which the oligosaccharide having a higher reducing power [32].

#### 2.4.3. Total Antioxidant Capacities of *P. notoginseng* Polysaccharides

The mechanism of the FRAP method for determining total antioxidant capacity is that under acidic conditions, antioxidants can reduce ferric tripyridyltriazine (Fe^3+^-TPTZ) to produce blue Fe^2+^-TPTZ, which can be detected at 593 nm [33]. As shown in Figure 7c, the total antioxidant capacity of *P. notoginseng* polysaccharides was in a dose-dependent manner. In general, the PP7 with EC_50_ of 1.92 mg/mL showed the strongest antioxidant capacity compared with the three oligosaccharides, which might be due to the high content of glucuronic acid in PP7.

### 2.5. The Effect of P. notoginseng Polysaccharides on Cells under Oxidative Stress

#### 2.5.1. *P. notoginseng* Polysaccharides Protected Cells against Oxidative Stress

In order to avoid the toxicity of the high concentration of four polysaccharides in HHL-5 cells, the appropriate polysaccharide concentration was estimated. As shown in Supplementary Figure S3, when the concentration was lower than 400 μg/mL, the four *P. notoginseng* polysaccharides had no toxic effect on the growth of HHL-5. When the concentration was higher than 400 μg/mL, all four polysaccharides exhibited a certain inhibitory effect on cell activity, which might be because the high concentration of polysaccharides increased the osmotic pressure in the culture medium.

**Figure 8:**
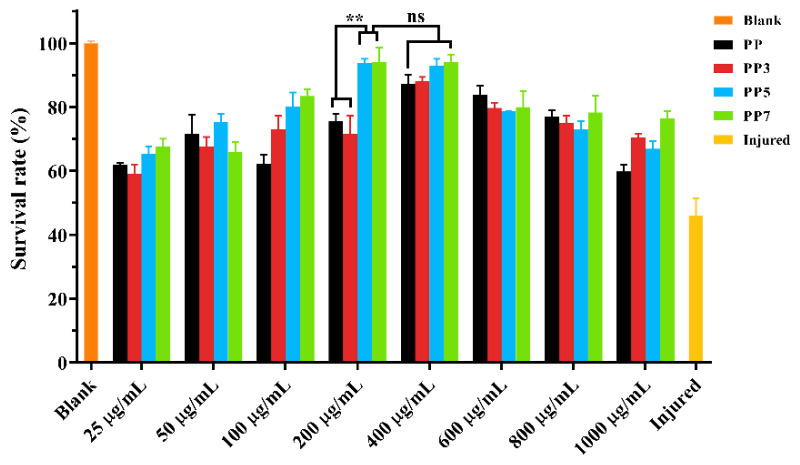
Treatment of PP, PP3, PP5, and PP7 significantly increased the oxidative stress resistance in HHL-5 cells. The values are presented as the mean ± SD (*n* = 3). **: Statistically significant differences between compared samples (*p* < 0.01), and the “ns” means no significant difference.

In the cells, free radicals are constantly generated and eliminated in the normal metabolic process. However, disrupted redox homeostasis induces excessive ROS generation, leading the oxidative damage to proteins, nucleotides, and other biological macromolecules in cells. To measure the antioxidant property of PP and its three oligosaccharides in the cell model, juglone was used to induce oxidative stress in HHL-5 cells by disrupting the electron transport chain in mitochondria, resulting in cell proliferation inhibition and apoptosis. As shown in Figure 8, the cell viability of the injury group (treated with juglone only) was 46.07%. After pretreatment with polysaccharides for 24 h, 50 to 400 μg/mL of all four samples could improve the cell survival rate in varying degrees under oxidative stress, while 200 and 400 μg/mL of PP5 and PP7 had the highest survival rate. Treatment of 400 μg/mL PP5 and PP7 increased the survival rate by 102.95% and 104.01%, compared to injure group. The results indicated that all four polysaccharides could alleviate the oxidative damage of cells, and the activities might be improved by the Fenton reaction. There was no significant difference in cell survival rate at 200 and 400 μg/mL. Therefore, 200 μg/mL was used as the subsequent experimental concentration.

#### 2.5.2. *P. notoginseng* Polysaccharides Could Alleviate Apoptosis by Inhibiting the ROS Production

**Figure 9:**
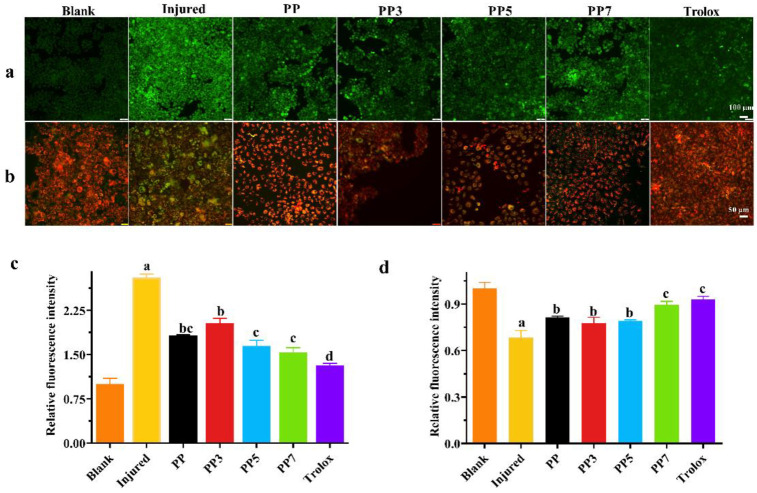
Treatment of PP, PP3, PP5, and PP7 significantly alleviates apoptosis by inhibiting intracellular ROS levels in HHL-5 cells, and different letters in the picture indicate significance, *p* < 0.05. (**a**) The depolarization of the mitochondria membrane induced the release of JC-1 from mitochondria to form JC-1 monomers. (**b**) JC-1 accumulated in mitochondria as JC-1 aggregates when the mitochondria membrane was intact. (**c**) The intracellular ROS contents in HHL-5 cells under juglone-induced oxidative stress. (**d**) Quantitative evaluation of JC-1 aggregates/monomers ratio.

ROS plays an important role in signal transduction. But the excessive accumulation of ROS will induce oxidative stress, resulting in cell apoptosis. As shown in Figure 9c, the ROS level in the injury group was significantly higher than in the blank group. After 24 h treatment with 200 μg/mL polysaccharide, the ROS levels of PP, PP3, PP5, and PP7 were significantly decreased by 34.98%, 27.65%, 41.34%, and 45.27%, respectively. Those results indicated that *P. notoginseng* polysaccharides could reduce the content of ROS in cells under juglone-induced oxidative stress.

Apoptosis plays a crucial role in maintaining cell homeostasis and acts as a defense mechanism against exogenous or endogenous stress. The change of mitochondrial membrane potential (MMP) is an early detection index of apoptosis. In this study, the fluorescent dye JC-1 was used to reveal the dynamic changes in mitochondrial. When the mitochondrial membrane potential is high, JC-1 aggregates in the matrix of mitochondria to form J-aggregates, which can produce red fluorescence (Figure 9b). When the mitochondrial membrane potential is low, JC-1 is a monomer in the cytoplasm, resulting in green fluorescence (Figure 9a). The transformation of JC-1 from red to green fluorescence indicates that the cell membrane potential decreases and the cell is in the early stage of apoptosis. As shown in Figure 9d, compared with the blank control, the ratio of red fluorescence to green fluorescence density in the injury group showed that the fluorescence intensity of MMP decreased significantly from 1.0 to 0.68. The results showed that juglone destroyed the mitochondrial membrane potential through oxidative stress. The treatment of PP, PP3, PP5, and PP7 increased the MMP by 13.19%, 9.53%, 10.97%, and 21.39%, respectively, indicating that the four polysaccharides could recover the mitochondrial membrane potential decreased by oxidative damage.

Compared to nature polysaccharides PP, PP7 exhibited significantly higher activities to decrease the intracellular ROS and juglone-induced apoptosis in HHL-5 cells, resulting in enhanced oxidative stress resistance. Those effects might be due to the reduction of Mw. After the Fenton reaction, the degraded polysaccharides PP7 might present a non-compact structure exposing more free hydroxyl groups to react with free radicals, which caused the increase in antioxidant activity [31]. Similar results were reported in degraded mucilage polysaccharides from *Dioscorea opposita*. Under the treatment of H_2_O_2_ and Vc, the Mw of *D. opposita* polysaccharide was decreased, while the antioxidant activities were significantly increased [34]. In addition, the degraded oligosaccharides were regarded to have a better uptake rate by cancer cells than impact polysaccharides. The increased bioavailability might be another reason for improved antioxidant activity in cells [35].

## 3. Materials and Methods

### 3.1. Materials and Reagents

*Panax notoginseng* leaves were collected in October 2021 in Wenshan Zhuang and Miao Autonomous Prefecture, Yunnan Province (23° N, 104° W) and identified by Professor Shiling Feng, School of life sciences, Sichuan Agricultural University.

Petroleum ether (60–90 °C), ferrous sulfate (FeSO_4_·7H_2_O), hydrogen peroxide (H_2_O_2_), glucose, phenol, sulfuric acid, water-free ethanol, DMSO (dimethyl sulfoxide) and ascorbic acid (VC) Trolox were all purchased from the Chengdu Kelong reagent factory. 1,1-Diphenyl-2-picrylhydrazy (DPPH), juglone, Trolox, and tripyridine triazine (TPTZ) were purchased from Sigma-Aldrich Chemical Company (St. Louis., MO, USA). All reagents were analytical grade. Fetal bovine serum (FBS), DMEM high-glucose medium, digestive enzymes, and phosphate-buffered saline (PBS) were bought from Hyclone Company (Logan, UT, USA).

### 3.2. Optimization of Extraction from P. notoginseng Polysaccharides

**Figure 10:**
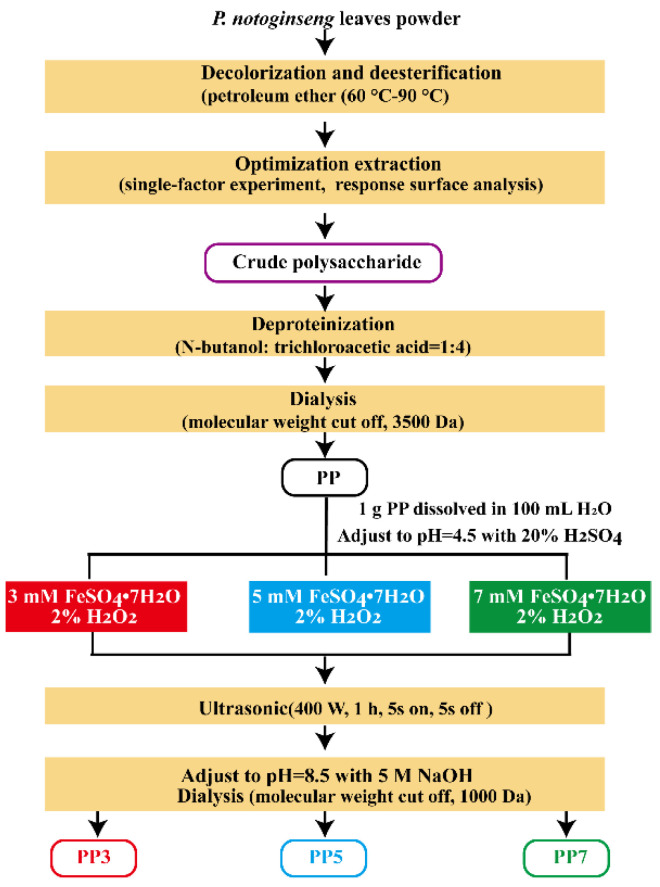
The extraction process and Fenton degradation reaction of polysaccharides from *P. notoginseng* leaves.

As shown in Figure 10, *P. notoginseng* leaves polysaccharides (PP) were extracted by hot water extraction. Briefly, the PP was obtained by hot-water extraction at different temperatures (50, 65, 75, 85 and 95 °C), liquid:solid ratios (10, 20, 30, 40, and 50 mL/g) and extraction times (0.5, 1.0, 1.5, 2.0 and 2.5 h). The supernatant was collected, concentrated, and precipitated with ethanol. The extraction yield was calculated as follows:

(2) $$Y\;(\%)=\frac{m_{1}}{m_{2}}\;\times \;100\%\;$$

where *m*_1_ is the weight of PP; *m*_2_ is the weight of the dry powder of *P. notoginseng* leaves.

The extraction process was optimized by performing a three-level-three factor Box Behnken Design (BBD) with RSM. Table 1 lists the coding and actual level of each factor. The average values of the three relevant parameters obtained by measurement are fitted to the second-order polynomial model.

### 3.3. Preparation of PP Oligosaccharides by Fenton Reaction

Oligosaccharides were prepared using the Fenton reaction with ferrous sulfate (FeSO_4_·7H_2_O) and hydrogen peroxide (H_2_O_2_) at a ratio of 1:1 (*v*/*v*) according to the described method with some modifications [36]. In brief, 1 g of PP was dissolved in 100 mL of deionized water, and the pH was adjusted to 5.0 by using 20% H_2_SO_4_ (*v*/*v*). FeSO4 was added to obtain the final concentration were 3.0, 5.0, and 7.0 mM, respectively. The Fenton reaction was started by adding an equal volume of 2% H_2_O_2_. The reaction was carried out in an ice bath for 1 h under an ultrasonic pulse (5 s off, 5 s on) with a total power of 400 W. The reaction was terminated by 5.0 M NaOH to adjust the pH value of the reaction mixture to 8.5. The mixture was then centrifuged at 3500× *g* rpm for 10 min to precipitate ferric ions and remove residual H_2_O_2_. The degraded products were concentrated, dialyzed (Molecular weight cutoff, 1000 Da), and lyophilized (Figure 10). Three oligosaccharides (PP3, PP5, and PP7) were obtained from different Fenton reaction systems.

### 3.4. Structural Properties

#### 3.4.1. Molecular Weight Distribution

The molecular weight and molecular configuration of PP and its degradation products were determined by high-performance size exclusion chromatography with differential multi-angle laser light scattering and refractive index detector (HPSEC-MALLS-RI, Thermo, Madison, WI, USA) equipped with a gel exclusion column (Ohpak sb-805 HQ 300 × 8 mm). Briefly, the samples were dissolved in 0.1 M NaNO_3_ aqueous solution. 0.1 M NaNO_3_ containing 0.02% (*w*/*v*) sodium azide was used as the mobile phase with a flow rate of 0.4 mL/min. Dextrans (Sigma-Aldrich, St. Louis, MO, USA) with different molecular weights (0.5 to 670 kDa) were used as calibration standards.

#### 3.4.2. Monosaccharide Compositions

The monosaccharide composition was determined by 1-phenyl-3-methyl-5-pyrazolone (PMP) pre-column derivatization-HPLC according to described methods with some modifications [37]. Briefly, 5.0 mg of samples were hydrolyzed with 4.0 mL of 2.0 M trifluoroacetic acid (TFA) at 100 °C for 6 h in an ampoule bottle. Residual trifluoroacetic acid was removed by adding NaOH. The hydrolysate of polysaccharide samples and monosaccharide standards solution was incubated with 0.5 M PMP and 0.3 M NaOH at 70 °C for 40 min. After incubation, 0.3 M HCl was added to neutralize excess alkali, and the mixture was extracted with chloroform 3 times. PMP-labeled samples were analyzed by a 1260 Agilent HPLC system (Agilent, Santa Clara, CA, USA) with Zorbax SB-C18 column (150 × 4.6 mm, 5 μm). The mobile phase was phosphate buffer (0.1 M, pH 6.85) and acetonitrile (82:18, *v*/*v*) at a flow rate of 1.0 mL/min. The mixture of different monosaccharides (glucose, rhamnose, galactose, mannose, arabinose, and xylose) was used as the standard.

#### 3.4.3. Fourier Transform Infrared (FT-IR)

The FT-IR spectra of all polysaccharides were obtained by Nicolet S10 instrument (Thermo Fisher Scientific, Waltham, MA, USA). 1 mg of sample was mixed with about 100 mg of potassium bromide, ground, and pressed into a pellet. The scanning frequency range was 4000–400 cm^−1^.

#### 3.4.4. Nuclear Magnetic Resonance (NMR) Spectroscopy

PP and its three degradable oligosaccharides were dissolved in D_2_O (0.5 mL) at 85 °C and lyophilized three times. The 1 H NMR spectra were collected on a Bruker AVANCE III 500 MHz spectrometer (Bruker, Rheinstetten, Germany) at 600 MHz.

#### 3.4.5. Thermal Characterization

Thermal analysis of four samples was investigated using differential scanning calorimetry (DSC) on a simultaneous thermal analyzer (STA 449 F3 Jupiter, Netzach, Selbu, Germany). Samples (5.0 mg) were heated from room temperature to 400 ºC at a rate of 10 ºC/min in a flowing argon atmosphere.

#### 3.4.6. X-ray Diffraction (XRD) Measurements

XRD study was carried out using an X’Pert PRO X-ray diffractometer (PANalyticals, Netherlands) and measured the four samples according to the method described by Wang et al. [29]; PP and its 3 degradable oligosaccharides were ground to a particle size of about 5 μm, used to fill the groove of the sample holder, and put into the sample table. The samples were scanned at a range of 5–80° 2θ. Data were collected with a step size of 0.02° 2θ at 25 °C. The scan time was 20 s for each step.

### 3.5. Antioxidant Activity

#### 3.5.1. DPPH· Scavenging Activity Assay

The DPPH· scavenging activities of PP and its three degradable oligosaccharides were measured according to the method described by Yeung et al. [32]. Briefly, 100 μL samples solution of different concentrations (0.25, 0.5, 0.2, 1.0, 2.0, 3.0, 4.0, and 5.0 mg/mL) and 100 μL DPPH solution (0.5 mM DPPH in ethanol) was mixed in the dark at 37 °C for 0.5 h. The absorbance of the mixture was measured at 517 nm (Beckman UV–Vis spectrophotometer, Pasadena, CA, USA). The DPPH· scavenging effect was calculated using the following formula:

(3) $$\mathrm{DPPH}\cdot \;\mathrm{scavenging}\;\mathrm{activity}\;(\%)=(1-\frac{A_{sample}-A_{blank}}{A_{control}})\;\times \;100\%$$

where A_control_, A_sample_ and A_blank_ are the absorbances of the polysaccharide with deionized water mixture, the polysaccharide solution containing DPPH·, and the polysaccharide solution without DPPH·, respectively.

#### 3.5.2. Reducing Power Assay

The reduction powers of *P. notoginseng* polysaccharides were determined by the potassium ferricyanide reduction method [38]. The four solutions with different concentrations were mixed with 2.0 M PBS buffer (pH 6.6) and 1% potassium ferricyanide solution for 1:1:1 (*v*/*v*) at 50 °C. After 20 min, 1.0 mL of trichloroacetic acid solution was added, and the supernatants were collected by centrifugation at 4000× *g* rpm for 10 min. The collected supernatant was mixed with 0.5 mL of 0.1% ferric chloride solution and 2.5 mL of ultrapure water. The absorbance of the mixture was recorded at 700 nm.

#### 3.5.3. Total Antioxidant Capacity Assay

The total antioxidant capacity assay was measured by ferric ion-reducing antioxidant power (FRAP) with a previous method [39]. In brief, 100 μL of *P. notoginseng* polysaccharides solutions with different concentrations were incubated with 500 μL FRAP reagent (10 mM TPTZ, 20 mM FeCl_3_, and 300 mM acetate buffer) for 10 min in the dark. The absorbance of the mixture was recorded at 593 nm. The total antioxidant capacity was expressed by the Trolox equivalent (TE µmol/100g).

### 3.6. Cell Viability Assay

#### 3.6.1. Maintenance of Cells

HHL5 cells were cryopreserved by the Department of Botany, College of life sciences, Sichuan Agricultural University. HHL5 cells were cultured in DMEM medium containing 10% FBS at 37 °C with 5% CO_2_. When the cell confluence reached 80%, the cells were digested from the flask and washed twice with PBS for subsequent analysis.

#### 3.6.2. Determination of *P. notoginseng* Polysaccharides Toxicity by MTT

The cell density was adjusted to 10^5^/mL, and 90 μL cell suspension was added to 96-well plates. After 12 h, 10 μL of *P. notoginseng* polysaccharides with different concentrations (0, 25, 50, 100, 200, 400, 600, 800, and 1000 μg/mL) were added to treat the HHL5 cells for 24 h. 100 μL fresh medium containing MTT solution (5.0 mg/mL) was added to form the formazan crystals. Finally, a volume of 100 μL DMSO was added to dissolve the formazan crystals after the medium was removed and the absorbance of the solution at 570 nm. The cell viability was calculated according to the following formula:

(4) $$\mathrm{Cell}\;\mathrm{viability}=\frac{A_{1}}{A_{2}}\;\times \;100\%$$

where *A*_1_ is the absorbance of the group treated with *P. notoginseng* polysaccharides and *A*_2_ is the absorbance of the blank group treated with the same volume of PBS.

#### 3.6.3. Protective Effect of *P. notoginseng* Polysaccharides on HHL-5 Cells under Oxidative Stress

After 24 h of cell culture, the *P. notoginseng* polysaccharides with different concentrations treated the HHL5 cells, and then 10 μmol/L of juglone was added to induce oxidative stress for 6 h. Finally, DMSO was added to dissolve the formazan crystals after the medium was removed and the absorbance of the solution at 570 nm. The cell viability was calculated according to the former Formula (2).

#### 3.6.4. Fluorescence Staining Assay

The cell density was adjusted to 10^6^ cells/mL and inoculated into 6-well plates for 24 h. the *P. notoginseng* polysaccharides (200 μg/mL) were added to each well to treat the HHL5 cells for 24 h. 10 μmoL/L of juglone was added to induce oxidative stress for 4 h. Then, the cells were fixed with paraformaldehyde at 4 °C for 2 h. H2DCF-DA, JC-1, and annexin V-PI were added to the culture plates for 30 min to measure the ROS level, mitochondrial membrane potential (MMP), and apoptosis under a fluorescence microscope (Olympus BX53, Tokyo, Japan).

#### 3.6.5. Statistical Analysis

All experiments were performed in at least three replicates. Statistical analyses were performed by GraphPad Prism9 software (GraphPad Software, San Diego, CA, USA). Statistical comparisons between treated and untreated cells were analyzed by one-way ANOVA and multiple comparisons.

## 4. Conclusions

In this study, PP was extracted using hot water extraction from *Panax notoginseng* leaves. Ultrasonic-assisted Fenton reaction was applied to generate PP-derived oligosaccharide fractions. After degradation, the Mw values of the PP in the degraded products were remarkably decreased from 64.69/14.64 kDa to 8.92, 5.99, and 5.89 kDa. Monosaccharide composition, FT-IR, and NMR analysis of four samples exhibited that the primary backbone characteristics were preserved after Fenton degradation. In addition, the XRD and thermal analysis showed that depolymerized oligosaccharide fractions had similar crystallinity structure and thermal stability. Furthermore, Fenton degradation improved the total antioxidant capacities of PP7. And the ability to alleviate oxidative stress in the cell of PP7 could be significantly enhanced after the Fenton reaction. These results suggested that ultrasonic-assisted Fenton reaction might be used to modify the natural polysaccharides with better biological activities.

## Figures and Tables

**Table 1:** Box-Behnken test design and results.

Test Number	Extraction Temperature (°C, A)	Extraction Time (h, B)	Extraction Liquid-Solid Ratio (mL/g, C)	Polysaccharide Yield (%)
1	0 (85)	0 (1.5)	0 (15)	12.01
2	0 (85)	1(2)	1 (20)	8.36
3	−1 (75)	0 (1.5)	1 (20)	8.58
4	0 (85)	0 (1.5)	0 (15)	11.56
5	−1 (75)	−1 (1)	0 (15)	8.14
6	1 (95)	−1 (1)	0 (15)	9.68
7	0 (85)	0 (1.5)	0 (15)	10.98
8	0 (85)	−1 (1)	−1 (0)	8.58
9	1 (95)	1 (2)	0 (15)	11.44
10	−1 (75)	1 (2)	0 (15)	8.58
11	1 (95)	0 (1.5)	−1 (10)	10.12
12	0 (85)	1 (2)	−1 (10)	9.24
13	0 (85)	−1 (1)	1 (20)	9.02
14	0 (85)	0 (1.5)	0 (10)	11.76
15	−1 (75)	0 (1.5)	−1 (10)	9.02
16	1 (95)	0 (1.5)	1 (20)	9.02
17	0 (85)	0 (1.5)	0 (15)	11.46

**Table 2:** The ANOVA of RSM results.

Variance Source	Sum of Squares	df	Mean-Square	*p*-Value	*F*-Value
Model	27.9	9	3.09	9.13	<0.01 **
A	4.41	1	4.41	13.04	<0.01 **
B	0.61	1	0.61	1.79	0.2229
C	0.49	1	0.49	1.45	0.2678
AB	0.44	1	0.44	1.29	0.2938
AC	0.11	1	0.11	0.32	0.5881
BC	0.44	1	0.44	1.29	0.2938
A^2^	3.07	1	3.07	9.09	0.0195 *
B^2^	6.47	1	6.47	19.13	<0.01 **
C^2^	9.66	1	9.66	28.56	<0.01 **
Residual	2.37	7	0.34		
Lack of fit	1.78	3	0.59	4.03	0.1058
Pure error	0.59	4	0.15		
Correlation total	30.16	16			

R^2^ = 0.9215; R^2^_adj_ = 0.8206; CV% = 2.58%; *: Means significant difference (*p* < 0.05); **: Means extremely significant difference (*p* < 0.01).

**Table 3:** Chemical component, monosaccharide composition molecular weigh of PP, PP3, PP5, and PP7.

Term	Monosaccharide	PP	PP3	PP5	PP7
Total sugar (%)		73.32 ± 3.15	81.63 ± 2.72	84.29 ± 2.98	85.82 ± 2.15
Yield (%)		11.30 ± 0.73	27.70 ± 0.65	32.89 ± 1.89	24.74 ± 0.54
Protein (%)		3.77 ± 0.08 ^a^	2.20 ± 0.02 ^b^	2.32 ± 0.12 ^b^	2.74 ± 0.05 ^b^
polyphenol (%)		8.72 ± 0.14 ^a^	2.50 ± 0.05 ^b^	1.96 ± 0.06 ^c^	1.39 ± 0.16 ^d^
Monosaccharide composition (mol%)	Man	5.35	8.13	8.84	
	Glc-UA	4.69	2.00	2.04	8.81
	Glc	24.79	29.30	21.71	27.92
	Gal	13.80	16.47	19.75	16.39
	Xyl	20.80	20.34	22.30	22.12
	Ara	27.28	23.76	25.37	24.76
	Fuc	3.29			
Mw (kDa)		64.69/14.64	8.92	5.99	5.89
Mn		50.92/6.76	3.45	2.99	2.68
PDI		1.27/2.17	2.59	2.14	2.19

Different letters in the table indicate significance, *p* < 0.05.

## Data Availability

The data set generated in this article can be provided to the corresponding author upon request.

## Supplementary Materials

The following supporting information can be downloaded at: https://www.mdpi.com/article/10.3390/ijms24054506/s1.

## References

[1] Wang T., Guo R., Zhou G., Zhou X., Kou Z., Sui F., Li C., Tang L., Wang Z. (2016). Traditional uses, botany, phytochemistry, pharmacology and toxicology of *Panax notoginseng* (Burk.) F.H. Chen: A review. J. Ethnopharmacol..

[2] Feng S., Cheng H., Xu Z., Yuan M., Huang Y., Liao J., Yang R., Zhou L., Ding C. (2018). *Panax notoginseng* polysaccharide increases stress resistance and extends lifespan in *Caenorhabditis elegans*. J. Funct. Foods.

[3] Hawthorne B., Lund K., Freggiaro S., Kaga R., Meng J. (2022). The mechanism of the cytotoxic effect of Panax notoginseng extracts on prostate cancer cells. Biomed. Pharmacother..

[4] Feng S., Cheng H., Xu Z., Shen S., Yuan M., Liu J., Ding C. (2015). Thermal stress resistance and aging effects of *Panax notoginseng* polysaccharides on *Caenorhabditis elegans*. Int. J. Biol. Macromol..

[5] Huang C., Jing X., Wu Q., Ding K. (2021). Novel pectin-like polysaccharide from *Panax notoginseng* attenuates renal tubular cells fibrogenesis induced by TGF-β. Carbohydr. Polym..

[6] Wang C., Zheng L., Liu S., Guo X., Qu Y., Gao M., Cui X., Yang Y. (2020). A novel acidic polysaccharide from the residue of *Panax notoginseng* and its hepatoprotective effect on alcoholic liver damage in mice. Int. J. Biol. Macromol..

[7] Shen S., Zhou C., Zeng Y., Zhang H., Hossen M.A., Dai J., Li S., Qin W., Liu Y. (2022). Structures, physicochemical and bioactive properties of polysaccharides extracted from *Panax notoginseng* using ultrasonic/microwave-assisted extraction. LWT Food Sci. Technol..

[8] Chan M.K., Yu Y., Wulamu S., Wang Y., Wang Q., Zhou Y., Sun L. (2020). Structural analysis of water-soluble polysaccharides isolated from *Panax notoginseng*. Int. J. Biol. Macromol..

[9] Feng S., Cheng H., Xu Z., Feng S., Yuan M., Huang Y., Liao J., Ding C. (2019). Antioxidant and anti-aging activities and structural elucidation of polysaccharides from *Panax notoginseng* root. Process Biochem..

[10] Liu S., Yang Y., Qu Y., Guo X., Yang X., Cui X., Wang C. (2020). Structural characterization of a novel polysaccharide from *Panax notoginseng* residue and its immunomodulatory activity on bone marrow dendritic cells. Int. J. Biol. Macromol..

[11] Wu D., He Y., Fu M., Gan R., Hu Y., Peng L., Zhao G., Zou L. (2022). Structural characteristics and biological activities of a pectic-polysaccharide from okra affected by ultrasound assisted metal-free Fenton reaction. Food Hydrocoll..

[12] Qiu J., Zhang H., Wang Z. (2019). Ultrasonic degradation of Polysaccharides from *Auricularia auricula* and the antioxidant activity of their degradation products. LWT Food Sci. Technol..

[13] Liu H., Lu X., Hu Y., Fan X. (2020). Chemical constituents of *Panax ginseng* and *Panax notoginseng* explain why they differ in therapeutic efficacy. Pharmacol. Res. Off. J. Ital. Pharmacol. Soc..

[14] Jin M.-Y., Li M.-Y., Huang R.-M., Wu X.-Y., Sun Y.-M., Xu Z.-L. (2021). Structural features and anti-inflammatory properties of pectic polysaccharides: A review. Trends Food Sci. Technol..

[15] Li D.Q., Li J., Dong H.L., Li X., Zhang J.Q., Ramaswamy S., Xu F. (2021). Pectin in biomedical and drug delivery applications: A review. Int. J. Biol. Macromol..

[16] Li J., Li S., Zheng Y., Zhang H., Chen J., Yan L., Ding T., Linhardt R.J., Orfila C., Liu D. (2019). Fast preparation of rhamnogalacturonan I enriched low molecular weight pectic polysaccharide by ultrasonically accelerated metal-free Fenton reaction. Food Hydrocoll..

[17] Xue H., Li P., Bian J., Gao Y., Sang Y., Tan J. (2022). Extraction, purification, structure, modification, and biological activity of traditional Chinese medicine polysaccharides: A review. Front. Nutr..

[18] Zhang N., Yang B., Mao K., Liu Y., Chitrakar B., Wang X., Sang Y. (2022). Comparison of structural characteristics and bioactivity of *Tricholoma mongolicum* Imai polysaccharides from five extraction methods. Front. Nutr..

[19] Shi X., Huang J., Wang S., Yin J., Zhang F. (2022). Polysaccharides from *Pachyrhizus erosus* roots: Extraction optimization and functional prope rties. Food Chem..

[20] Zhou L., Luo S., Li J., Zhou Y., Wang X., Kong Q., Chen T., Feng S., Yuan M., Ding C. (2021). Optimization of the extraction of polysaccharides from the shells of *Camellia oleifera* and evaluation on the antioxidant potential in vitro and in vivo. J. Funct. Foods.

[21] Wang Q., Xing N., Zhang Z., Peng D., Li Y., Wang X., Wang R., He Y., Zeng Y., Kuang H. (2021). Optimization of steaming process for polysaccharides from *Panax notoginseng* by box-behnken response surface methodology and comparison of immunomodulatory effects of raw and steamed *Panax notoginseng* polysaccharides. Pharmacogn. Mag..

[22] Wu J., Chen R., Tan L., Bai H., Tian L., Lu J., Gao M., Bai C., Sun H., Chi Y. (2022). Ultrasonic disruption effects on the extraction efficiency, characterization, and bioactivities of polysaccharides from *Panax notoginseng* flower. Carbohydr. Polym..

[23] Jiang Y., Ran J., Mao K., Yang X., Zhong L., Yang C., Feng X., Zhang H. (2022). Recent progress in Fenton/Fenton-like reactions for the removal of antibiotics in aqueous environments. Ecotoxicol. Environ. Saf..

[24] Huang L., Zhao J., Wei Y., Yu G., Li F., Li Q. (2021). Structural characterization and mechanisms of macrophage immunomodulatory activity of a pectic polysaccharide from *Cucurbita moschata* Duch. Carbohydr. Polym..

[25] Wang L., Zhao Z., Zhao H., Liu M., Lin C., Li L., Ma B. (2022). Pectin polysaccharide from *Flos magnoliae* (Xin Yi, *Magnolia biondii Pamp*. flower buds): Hot-compressed water extraction, purification and partial structural characterization. Food Hydrocoll..

[26] Zhang Z., Wang X., Mo X., Qi H. (2013). Degradation and the antioxidant activity of polysaccharide from *Enteromorpha linza*. Carbohydr. Polym..

[27] Combo A.M., Aguedo M., Quievy N., Danthine S., Goffin D., Jacquet N., Blecker C., Devaux J., Paquot M. (2013). Characterization of sugar beet pectic-derived oligosaccharides obtained by enzymatic hydrolysis. Int. J. Biol. Macromol..

[28] Zhou S., Huang G., Huang H. (2022). Extraction, derivatization and antioxidant activities of onion polysaccharide. Food Chem..

[29] Wang W., Ma X., Jiang P., Hu L., Zhi Z., Chen J., Ding T., Ye X., Liu D. (2016). Characterization of pectin from grapefruit peel: A comparison of ultrasound-assisted and conventional heating extractions. Food Hydrocoll..

[30] Zhang F., Zhang X., Liang X., Wu K., Cao Y., Ma T., Guo S., Chen P., Yu S., Ruan Q. (2022). Defensing against oxidative stress in *Caenorhabditis elegans* of a polysaccharide LFP-05S from *Lycii fructus*. Carbohydr. Polym..

[31] Ahmadi S., Yu C., Zaeim D., Wu D., Hu X., Ye X., Chen S. (2022). Increasing RG-I content and lipase inhibitory activity of pectic polysaccharides extracted from goji berry and raspberry by high-pressure processing. Food Hydrocoll..

[32] Yeung Y.K., Kang Y.-R., So B.R., Jung S.K., Chang Y.H. (2021). Structural, antioxidant, prebiotic and anti-inflammatory properties of pectic oligosaccharides hydrolyzed from okra pectin by Fenton reaction. Food Hydrocoll..

[33] Zou Y.F., Zhang Y.Y., Paulsen B.S., Rise F., Chen Z.L., Jia R.Y., Li L.X., Song X., Feng B., Tang H.Q. (2020). Structural features of pectic polysaccharides from stems of two species of *Radix codonopsis* and their antioxidant activities. Int. J. Biol. Macromol..

[34] Zhang Z., Wang X., Liu C., Li J. (2016). The degradation, antioxidant and antimutagenic activity of the mucilage polysaccharide from *Dioscorea opposita*. Carbohydr. Polym..

[35] Kapoor S., Dharmesh S.M. (2017). Pectic Oligosaccharide from tomato exhibiting anticancer potential on a gastric cancer cell line: Structure-function relationship. Carbohydr. Polym..

[36] Li J., Li S., Liu S., Wei C., Yan L., Ding T., Linhardt R.J., Liu D., Ye X., Chen S. (2019). Pectic oligosaccharides hydrolyzed from citrus canning processing water by Fenton reaction and their antiproliferation potentials. Int. J. Biol. Macromol..

[37] Xu Z., Wang B., Fu L., Wang H., Liu J., Zhou L., Yuan M., Ding C. (2019). Optimization Extraction, Purification and Antioxidant Activities of Polysaccharides from *Penthorum Chinense* Pursh. Int. J. Food Eng..

[38] Cheng H., Feng S., Jia X., Li Q., Zhou Y., Ding C. (2013). Structural characterization and antioxidant activities of polysaccharides extracted from *Epimedium acuminatum*. Carbohydr. Polym..

[39] Feng S., Xu X., Tao S., Chen T., Zhou L., Huang Y., Yang H., Yuan M., Ding C. (2022). Comprehensive evaluation of chemical composition and health-promoting effects with chemometrics analysis of plant derived edible oils. Food Chem. X.

