# Supplementary material for: Extraction, Structural, and Antioxidant Properties of Oligosaccharides Hydrolyzed from Panax notoginseng by Ultrasonic-Assisted Fenton Degradation
Source: Int J Mol Sci. 2023 Feb 24;24(5):4506. doi: 10.3390/ijms24054506 (PMC10003133; doi:10.3390/ijms24054506)
Supplement: Supplementary file 1 [file ijms-24-04506-s001.zip › ijms-2209887-supplementary.pdf]

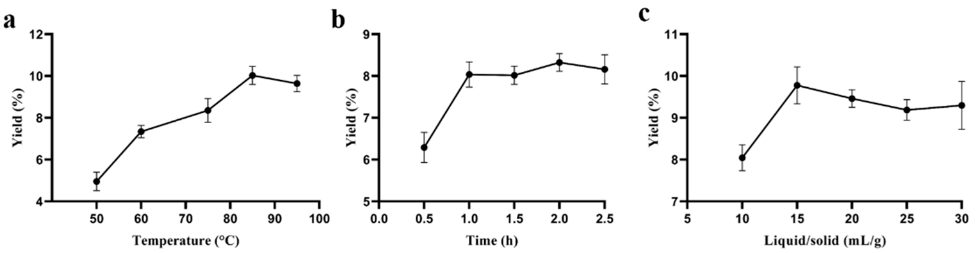

Figure S1. Effect of single factors on the yield of PP. Effect of (**a**) extraction temperature, (**b**) extraction time, and (**c**) liquid/solid ratio on the yield of PP.

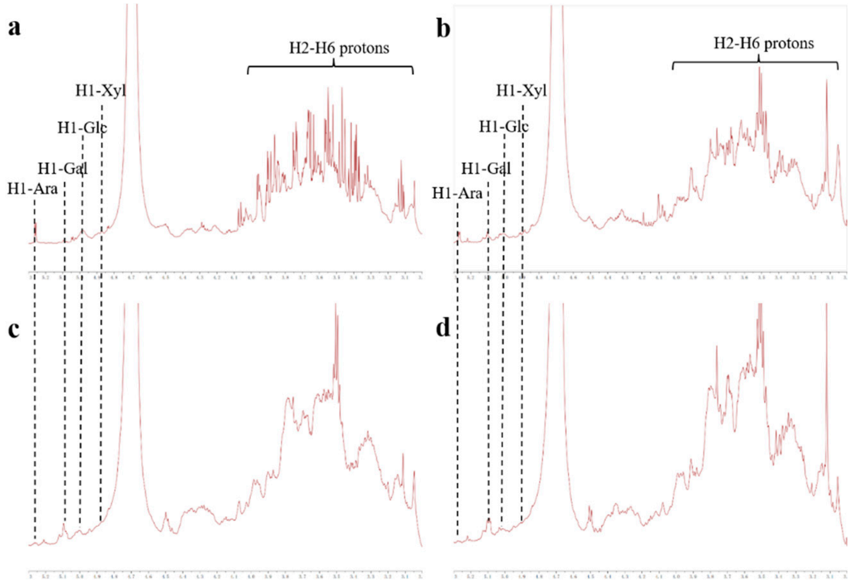

Figure S2. 1H NMR spectra of PP (**a**), PP3 (**b**), PP5 (**c**), and PP7 (**d**).

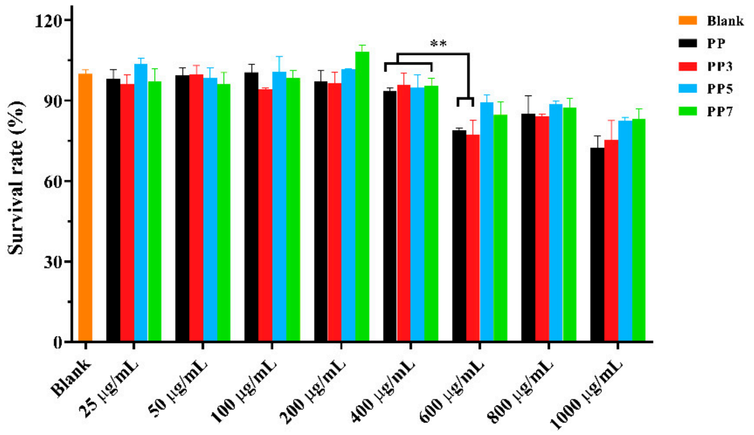

Figure S3. Cell viability of HHL-5 cells after the treatment of PPs with concentration from 25 to 1000 μg/mL. The value is presented as the mean ± SD (n = 3). **: Statistically significant differences between compared samples (*p < 0.01*).
